# Suicide risk among prisoners in French Guiana: prevalence and predictive factors

**DOI:** 10.1186/s12888-017-1320-4

**Published:** 2017-05-02

**Authors:** Gülen Ayhan, Romain Arnal, Célia Basurko, Vincent About, Agathe Pastre, Eric Pinganaud, Dominique Sins, Louis Jehel, Bruno Falissard, Mathieu Nacher

**Affiliations:** 1 Inserm CIC 1424, Centre d’Investigation Clinique Antilles Guyane, Centre Hospitalier de Cayenne, Avenue des Flamboyants, BP 6006, 97 306 Cayenne CEDEX, France; 2 Centre d’Investigation Clinique Antilles-Guyane, CIC INSERM 1424, Centre Hospitalier Andrée Rosemon, Cayenne, French Guiana France; 3 Unité de Soins et de Consultations Ambulatoires, Centre Hospitalier Andrée Rosemon, Cayenne, French Guiana France; 4 Équipe IPSOM, INSERM 1178, Paris, France; 5 Department of Psychiatry, Centre Hospitalier Universitaire de Martinique, Fort-de-France, Martinique France; 6 EA3593, Université de Guyane, Cayenne, French Guiana France

**Keywords:** Suicide, Prisons, Child abuse, Mental disorders, South America

## Abstract

**Background:**

Suicide rates in prison are high and their risk factors are incompletely understood. The objective of the present study is to measure the risk of suicide and its predictors in the only prison of multicultural French Guiana.

**Methods:**

All new prisoners arriving between September 2013 and December 2014 were included. The Mini International Neuropsychiatric Interview (MINI) was used and socio-demographic data was collected. In order to identify the predictors of suicide risk multivariate logistic regression was used.

**Results:**

Of the 707 prisoners included 13.2% had a suicidal risk, 14.0% of whom had a high risk, 15.1% a moderate risk and 41.9% a low risk. Predictive factors were depression (OR 7.44, 95% CI: 3.50–15.87), dysthymia (OR 4.22, 95% CI: 1.34–13.36), panic disorder (OR 3.47, 95% CI: 1.33–8.99), general anxiety disorder (GAD) (OR 2.19, 95% CI: 1.13–4.22), men having been abused during childhood (OR 21.01, 95%, CI: 3.26–135.48), having been sentenced for sexual assault (OR 7.12, 95% CI: 1.98–25.99) and smoking (OR 2.93, 95%, CI 1.30–6.63).

**Conclusion:**

The suicide risk was lower than in mainland France, possibly reflecting the differences in the social stigma attached to incarceration because of migrant populations and the importance and trivialization of drug trafficking among detainees. However, there were no differences between nationalities. The results reemphasize the importance of promptly identifying and treating psychiatric disorders, which were the main suicide risk factors.

## Background

According to the World Health Organisation (WHO), 800,000 persons in the general population commit suicide every year worldwide. The global standardized suicide rate is 11.4 for 100,000 habitants. WHO estimates that throughout the world every 3 s, a suicide attempt occurs, and that every minute a suicide is committed [1]. Although the mechanisms of suicide are not completely understood, some risk factors have been identified, such as being a young male, belonging to an indigenous population, suffering from mental disorders or alcohol and/or substance abuse, having a previous history of suicide attempt and being in custody [2].

Prisoners are a particularly vulnerable group with a nine fold increase of suicidal risk and a two-fold increase of suicide rate when compared to the general population [1]. This makes suicide the leading cause of death in inmates and a public health problem [1, 3]. Suicide in prison is not only a health concern because of its mortality but also because witnessing it has been identified as the most striking event during imprisonment [4].

Until today, there have been relatively few studies investigating suicide in prison. A British study revealed that the standardised mortality ratio for suicide was 5.1 (95% CI, 4.8–5.3) in English and Welsh prisons in reference to the general population [5]. France has the highest suicide rate among 15 European countries, with an increasing rate in the last 50 years increasing from 4/10000 in the 1960^s^ to 19/10000 in 2008, with a peak in 1996 of 26/10000. French inmates commit suicide 6 times more often than men in the general population [6]. Comparison of suicide rates worldwide seems to be complicated because of the differences of correctional conditions and inclusion criteria for suicides (whether committed after release, died in the hospital or in prison) [6].

Until today, there has been no significant correlation between overpopulation in prisons and the suicide rate, on the contrary, being alone in a cell is considered as a risk factor for suicide [7, 8]. In France suicide in prison occurs mainly at the beginning of incarceration, 25% during the first 2 months and 50 % in the first 6 months [9]. This is often explained by the incarceration shock which is related to the difficulty of adapting to prison, the deprivation of liberty, ostracism and the humiliation due to the disclosure of a committed crime in front of family and society [10, 11]. Mental illness seems to be an important factor in prison suicidality [12]. In Spain, suicidality in prison was associated with certain psychiatric diagnoses, including affective disorder, substance dependence disorders, personality disorders, anxiety disorder and a family psychiatric history [13]. A European supranational study identified sexual offenders, offenders charged with violent crimes and prisoners sentenced for short- and long-term imprisonment to be at an higher suicide risk [14].

In a multinational review, no correlation was observed between the general suicide rate in the population and suicide in correctional circumstances, thus highlighting the entity of suicide in prison and the need for further investigation [6].

Upon request of the French Government, a nationwide survey of the prevalence of psychiatric illnesses in prisons in France and in Martinique (French West Indies) was conducted in 2004. Among 799 inmates, 40.3% of those in France and 26% of those in Martinique had a suicide risk [4]. The identification of predictive factors for suicide risk was not aim of that study.

French Guiana is a French overseas territory in South America between Northern Brazil and Suriname. Being a European country with access to a structured social system and economic advantages, it has a large immigrant population. Its population is very heterogeneous in terms of socio economic categories and cultures. The Guianese population is composed of Creoles, Maroons, Surinamese, Brazilians, Guyanese, Chinese, Hmong people and French from continental France. This diversified composition permits the investigation of different ethnic groups in one territory. The correctional centre in French Guiana is the only prison in this region. It has a capacity of 650 inmates*.* In 2011–2015, there were five suicides (annual average 15.3/10000) whereas in continental France 113 suicides for 65,000 inmates (17.4/10000) occurred.

Suicidality in general has never been investigated in French Guiana. PubMed research gave no relevant result but just one case report of toxic plant ingestion in a suicide attempt [15].

The objective of this study was thus to evaluate the prevalence of suicidal risk and its predictive factors in the correctional population of French Guiana. Studies have been conducted in metropolitan France and in Martinique, but this was the first study in this particular context of French Guiana.

## Methods

In this cross-sectional study we included all consenting new adult prisoners incarcerated between 18/09/2013 and 31/12/2014 at the penitentiary centre of French Guiana, situated in Rémire-Montjoly, 10 km from the capital Cayenne. Inmates that were assigned a legal guardian were excluded. This decision was based on the fact that the required presence of the legal guardian would have been logistically very difficult given the restricted access to the mental health ward.

In the usual incarceration procedure, after passing administrational registration, all new arrivals are seen for physical examination by a doctor of the “Unité de consultation et de soins ambulatoires (UCSA) “, the ambulatory care unit of the prison and then by a psychiatrist or psychiatric nurse in the “Unité fonctionnelle de psychiatrie intra-carcérale (UFPI)“, the psychiatric ward. In addition to this normal admission process and for the purpose of our study we added the Mini International Neuropsychiatric Interview (MINI). The MINI is a short diagnostic structured interview (DSI) developed in France and the United States to explore 17 disorders according to the Diagnostic and Statistical Manual (DSM)-V diagnostic criteria. Its validity and reliability has been confirmed in several studies [16–21]. Its applicability has also been validated in different studies [22–24]. The MINI has been translated and validated in 46 languages [20]. It is structured to allow use by non-specialized interviewers for the research of current disorders and is today one of the most used psychiatric diagnostic tools [25]. For each pathology, one or two screening questions rule out the diagnosis when answered negatively. Hence, the MINI is adapted for epidemiological studies with a need for a short but robust tool. The estimated time for passing the interview is 15 min [19]. The questions which evaluate the suicide risk (Suffer any accident? Plan or intend to hurt yourself in that accident either passively or actively? Did you intend to die as a result of this accident? Think that you would be better off dead or wish you were dead? Want to harm yourself or to hurt or to injure yourself? Think about suicide? Have a suicide plan? Take any active steps to prepare to injure yourself or to prepare for a suicide attempt in which you expected or intended to die? Deliberately injure yourself without intending to kill yourself? Attempt suicide? Did you ever make a suicide attempt?) and define its severity are presented in Table 1 [19]. The authors of the MINI defined three levels of suicide risk (low, medium or high risk). The MINI suicide risk scale has been prospectively validated in Sweden showing its potential to identify those at risk of committing suicide [26].

**Table 1:** Questions of the MINI defining the suicide risk and its severity

In the past month did you:			
Item	Question	Answer	Points
C1	Suffer any accident?	NO YES	0
IF NO TO C1, SKIP TO C2; IF YES, ASK C1a,:			
C1a	Plan or intend to hurt yourself in that accident either passively or actively?	NO YES	0
IF NO TO C1a, SKIP TO C2: IF YES, ASK C1b,:			
C1b	Did you intend to die as a result of this accident?	NO YES	0
C2	Think that you would be better off dead or wish you were dead?	NO YES	1
C3	Want to harm yourself or to hurt or to injure yourself?	NO YES	2
C4	Think about suicide?	NO YES	6
IF YES, ASK ABOUT THE INTENSITY AND FREQUENCY OF THE SUICIDAL IDEATION:			
Frequency: Occasionally, Often, Very often			
Intensity: Mild, Moderate, Severe			
Can you control these impulses and state that you will not act on them while in this program? Only score 8 points if response is NO.		NO YES	8
C5	Have a suicide plan?	NO YES	8
C6	Take any active steps to prepare to injure yourself or to prepare for a suicide attempt in which you expected or intended to die?	NO YES	9
C7	Deliberately injure yourself without intending to kill yourself?	NO YES	4
C8	Attempt suicide? Hoped to be rescued/survive Expected/intended to die	NO YES	10
In your lifetime:			
C9	Did you ever make a suicide attempt?	NO YES	4
IS AT LEAST 1 OF THE ABOVE (EXCEPT C1) CODED YES? IF YES, ADD THE TOTAL NUMBER OF POINTS FOR THE ANSWERS (C1-C9) CHECKED ‘YES’ AND SPECIFY THE LEVEL OF SUICIDE RISK AS INDICATED IN THE DIAGNOSTIC BOX:	NO	YES	
	*SUICIDE RISK CURRENT*		
	1–8 points 9–16 points >17 points	Low Moderate High	
MAKE ANY ADDITIONAL COMMENTS ABOUT YOUR ASSESSMENT OF THIS PATIENT’S CURRENT AND NEAR FUTURE SUICIDE RISK IN THE SPACE BELOW:			

The MINI has been translated and validated for the main languages spoken in French Guiana: French, English, Portuguese, Dutch and Spanish. All psychiatrists and nurses performing the MINI were trained in order to use the questionnaire correctly. We included socio-demographic questions (age, birth place, residence, languages, presence of a translator, family status, children, siblings, position among siblings, professional situation), history of detention (reason for detention, previous imprisonment) and psychiatric history. A training period preceded our study in order to test the feasibility of the MINI and to familiarize the research staff with the protocol. Proficiency was verified before starting the study.

### Statistical analysis

For statistical analysis we used Stata13 (College Station, Texas, USA). After descriptive analysis of qualitative and quantitative variables, we performed bivariate analysis in order to identify significant variables for suicide risk among inmates. Variables with *p* < 0.20 were included in the multivariate model for logistic regression and backward stepwise elimination was performed in order to identify independent risk factors for suicide risk. Collinearity was ruled out by using the collin package (STATA, College Station, Texas) and verifying that variance inflation factors were <4. The Hosmer-Lemeshow goodness-of-fit test was used for the final model.

## Results

Between September 18th 2013 and December 31st 2014, 785 new prisoners were registered in the correctional facility in French Guiana. The survey participation rate was 90% (707/785). The majority were males 647/707 (91.5%) and the mean age was 30 years with a median of 27 years (18–71 years). Detainees were mostly born in French Guiana (47.8%), followed by Surinam (15.1%), Guyana (14.3%) and Brazil (10.6%). The remaining inmates were mainly born in Haiti, Martinique, Guadeloupe, and continental France. 54.4% of the prisoners spoke French, 13.6% English and 12.3% Nenge Tongo, the language of the Maroons.

**Fig. 1:**
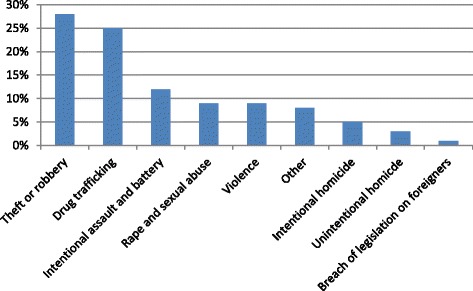
Motives of incarceration of the inmates with a suicide risk in the French Guianese Prison, Cayenne, 2013/2014 (*n* = 707)

The MINI revealed that 14.3% (101/707) of the inmates suffered from depression, 5.4% (38/707) had dysthymia, and 13.2% (93/707) had a suicidal risk, of which 14.0% (13/93) had a high risk, 15.1% (14/93) a moderate risk and 41.9% (39/93) a low risk of suicide. For 27 of those detainees the level could not be estimated for unknown reasons. Furthermore the MINI identified a maniac disorder in 3.7% (26/707) cases, a panic disorder in 6.8% (48/707), agoraphobia in 10.8% (76/707), social anxiety disorder (SAD) in 8.9% (63/707), obsessive compulsive disorder (OCD) in 2.1% (15/707), post-traumatic stress disorder (PTSD) in 15.1% (107/707), alcohol dependence in 17.5% (124/707), drug dependence in 33.2% (235/707), psychotic disorder in 7.2% (51/707), anorexia in 0.7% (5/707), bulimia in 0.4% (3/707), general anxiety disorder (GAD) in 25.7% (182/707) and antisocial personality disorder in 34.7% (245/707) cases. Among the inmates having a suicidal risk, the majority were men (87%), the mean age was 29 years, 74% were living in an urban area, 81% spoke French during the interview, 52% declared irregular odd jobs and 58% had previously been incarcerated (Table 2). Figure 1 describes for the motives of incarceration of the inmates with a suicide risk in our study population. Theft, robbery and drug trafficking were the main offenses. In the bivariate analysis, several past psychiatric conditions were significantly associated with suicide risk, depression having the highest Odds Ratio (OR 7.44; 95% CI 3.50–15.84). Childhood abuse (OR 20.37. 95% CI 5.27–78.77), especially for men (OR 26.74; 95% CI 5.44–131.52), was strongly associated with suicide risk. Other variables such as smoking and drug consumption were independently associated with suicide risk (Table 2). After multivariate analysis, having been sentenced for sexual abuse, smoking, being male, childhood abuse, depression, dysthymia, panic disorder and GAD remained significantly associated with the risk of suicide. After ruling analysis with the Stata collin package variance inflation factors ranged between 1.0 and 1.91, therefore there was no collinearity problem.

**Table 2:** Predictive factors of suicidal risk among detainees in the correctional centre of French Guiana, Cayenne 2013–2014

		Suicidal risk			Crude OR		Adjusted OR	
Variables		Yes *n (*%*)*	No *n* (%)	*p*-value	OR (95% CI)	*p*-value	OR (95% CI)	*p*-value
Gender								
	female (*n* = 60)	12 (0.20)	48 (0.80)	0.101	1.75 (0.89–3.43)	0.105	2.40 (0.85–6.77)	0.097
	male (*n* = 647)	81 (0.13)	566 (0.87)					
Age	(*n* = 707)	29	30	0.449	0.99 (0.97–1.01)	0.448	0.98 (0.94–1.01)	0.187
Number of children	(*n* = 704)	2.2	2.0	0.466	1.03 (0.95–1.12)	0.466		
Number of siblings	(*n* = 668)	5.5	5.3	0.633	1.01 (0.96–1.08)	0.633		
Position among siblings	(*n* = *n* = 288)	2.6	2.9	0.483	0.94 (0.78–5.13)	0.483		
Number of incarcerations	(*n* = 335)	2.4	2.1	0.391	1.05 (0.94–1.18)	0.395		
Domiciliation								
	Town (*n* = 423)	68 (0.74)	355 (0.59)	0.039*	2.51 (1.17–5.40)	0.018*	1.52 (0.52–4.40)	0.442
	Village (*n* = 23)	2 (0.02)	21 (0.03)		1.25 (0.25–6.30)	0.787	1.01 (0.11–8.96)	0.990
	Border town (*n* = 139)	14 (0.15)	125 (0.21)		1.47 (0.59–3.64)	0.405	0.76 (0.22–2.63)	0.662
	other (*n* = 113)	8 (0.09)	105 (0.17)				1	
First language other than French								
	yes (*n* = 292)	41 (0.44)	251 (0.41)	0.575	1.13 (0.73–1.76)	0.575		
	no (*n* = 413)	52 (0.56)	361 (0.59)					
Second language other than French								
	yes (*n* = 207)	29 (0.47)	178 (0.46)	0.951	1.02 (0.59–1.74)	0.951		
	no (*n* = 239)	33 (0.53)	206 (0.54)					
Third language other than French								
	yes (*n* = 146)	26 (0.84)	120 (0.67)	0.055	2.60 (0.95–7.11)	0.063		
	no (*n* = 65)	5 (0.16)	60 (0.33)					
Forth language other than French								
	yes (*n* = 49)	11 (0.92)	38 (0.70)	0.127	4.63 (0.55–38.93)	0.158		
	no (*n* = 17)	16 (0.30)	1 (0.08)					
Language spoken during interview								
	French (*n* = 563)	74 (0.81)	489 (0.80)	0.070	1.00			
	Brazilian (*n* = 37)	2 (0.02)	35 (0.06)		0.38 (0.89–1.60)	0.187		
	Spanish (*n* = 6)	2 (0.02)	4 (0.01)		3.30 (0.59–18.36)	0.172		
	English (*n* = 52)	9 (0.10)	43 (0.07)		1.38 (0.65–2.95)	0.402		
	Nenge Tongo (*n* = 36)	2 (0.02)	34 (0.06)		0.39 (0.09–1.65)	0.201		
	Dutch (*n* = 5)	2 (0.02)	3 (0.004)		4.41 (0.72–26.80)	0.108		
Employed								
	yes (*n* = 289)	46 (0.52)	243 (0.43)	0.110	1.44 (0.92–2.26)	0.111		
	no (*n* = 362)	42 (0.48)	320 (0.57)					
Previous incarceration								
	yes (*n* = 344)	54 (0.58)	290 (0.47)	0.051	1.55 (1.00–2.41)	0.053	1.04 (0.51–2.10)	0.918
	no (=363)	39 (0.42)	324 (0.53)					
Sentenced drug trafficking								
	yes (*n* = 243)	23 (0.25)	220 (0.36)	0.037*	0.59 (0.36–0.97)	0.038*	0.86 (0.38–1.95)	0.724
	no (=458)	69 (0.75)	389 (0.64)					
Sentenced for sexual assault								
	yes (*n* = 32)	8 (0.09)	24 (0.04)	0.042*	2.31 (1.01–5.32)	0.048*	7.12 (1.98–25.99)	0.003**
	no (*n* = 675)	85 (0.91)	590 (0.96)					
Smoking								
	yes(*n* = 383)	68 (0.77)	315 (0.56)	0.000***	2.71 (1.60–4.58)	0.000*	2.93 (1.30–6.63)	0.009**
	no(=271)	20 (0.23)	251 (0.44)					
Drug consumption								
	yes (*n* = 344)	59 (0.17)	285 (0.83)	0.004**	1.93 (1.22–3.06)	0.005**	1.00 (0.16–6.30)	0.997
	no (*n* = 331)	32 (0.10)	299 (0.90)					
Cannabis consumption								
	yes (*n* = 309)	53 (0.57)	256 (0.42)	0.006**	1.85 (1.19–2.88)	0.006**	0.87 (0.16–4.73)	0.870
	no (=398)	40 (0.43)	358 (0.58)					
Abused in childhood								
	yes (*n* = 11)	8 (0.11)	3 (0.01)	0.000***	20.37 (5.27–78.77)	0.000***		
	no (*n* = 553)	64 (0.89)	489 (0.99)					
Interaction gender/abuse in childhood								
	abused female (*n* = 2)	1 (0.01)	1 (0.002)	0.000***	7.64 (0.47–123.66)	0.152	0.44 (0.02–11.05)	0.618
	abused male (*n* = 9)	7 (0.10)	2 (0.004)		26.74 (5.44–131.52)	0.000***	21.01 (3.26–135.48)	0.001**
	never abused female/male(*n* = 553)	64 (0.89)	489 (0.99)				1	
Depression								
	yes (*n* = 101)	45 (0.46)	56 (0.55)	0.000***	9.34 (5.72–15.26)	0.000***	7.44 (3.50–15.87)	0.000***
	no (*n* = 606)	48 (0.08)	558 (0.92)				1	
Dysthymia								
	yes (*n* = 38)	19 (0.50)	19 (0.50)	0.000***	8.04 (4.07–15.87)	0.000***	4.22 (1.34–13.36)	0.014*
	no (*n* = 669)	74 (0.11)	595 (0.89)				1	
Manic								
	yes (*n* = 26)	8 (0.31)	18 (0.69)	0.014*	3.12 (1.31–7.39)	0.010*	0.39 (0.07–2.11)	0.277
	no (*n* = 681)	85 (0.12)	596 (0.88)				1	
Panic disorder								
	yes (*n* = 48)	17 (0.35)	31 (0.65)	0.000***	4.21 (2.22–7.96)	0.000***	3.47 (1.33–8.99)	0.011*
	no (*n* = 659)	76 (0.12)	583 (0.88)				1	
Agoraphobia								
	yes (*n* = 76)	17 (0.22)	59 (0.78)	0.012*	2.10 (1.17–3.80)	0.014*	0.74 (0.28–1.97)	0.549
	no (*n* = 631)	76 (0.12)	555 (0.88)				1	
SAD								
	yes (*n* = 63)	13 (0.21)	50 (0.79)	0.066	1.83 (0.95–3.52)	0.069	0.76 (0.27–2.09)	0.590
	no (*n* = 644)	80 (0.12)	564 (0.88)				1	
OCD								
	yes (*n* = 15)	6 (0.40)	9 (0.60)	0.008**	4.64 (1.61–13.34)	0.004**	2.08 (0.38–11.35)	0.397
	no (*n* = 692)	87 (0.13)	605 (0.87)				1	
PTSD								
	yes (*n* = 107)	34 (0.37)	73 (0.12)	0.000***	4.27 (2.62–6.95)	0.000***	2.00 (0.94–4.26)	0.073
	no (*n* = 600)	59 (0.63)	541 (0.88)				1	
Alcohol dependence								
	yes (*n* = 124)	18 (0.19)	106 (0.17)	0.621	1.15 (0.66–2.00)	0.621		
	no (*n* = 583)	75 (0.81)	508 (0.83)					
Drug dependence								
	yes (*n* = 235)	49 (0.53)	186 (0.30)	0.000***	2.56 (1.65–3.99)	0.000***	1.44 (0.60–3.45)	0.418
	no (*n* = 472)	44 (0.47)	428 (0.70)				1	
Psychosis								
	yes (*n* = 51)	17 (0.18)	34 (0.06)	0.000***	3.82 (2.03–7.16)	0.000***	1.12 (0.36–3.42)	0.847
	no (*n* = 656)	76 (0.82)	580 (0.94)					
Anorexia nervosa								
	yes (*n* = 5)	2 (0.02)	3 (0.005)	0.131	4.48 (0.74–27.15)	0.103		
	no (*n* = 702)	91 (0.98)	611 (0.995)					
Bulimia nervosa								
	yes (*n* = 3)	1 (0.01)	2 (0.003)	0.300	3.33 (0.30–37.05)	0.328		
	no (*n* = 704)	92 (0.99)	612 (0.997)					
GAD								
	yes (*n* = 182)	47 (0.51)	135 (0.22)	0.000***	3.63 (2.31–5.68)	0.000***	2.19 (1.13–4.22)	0.020*
	no (*n* = 525)	46 (0.49)	479 (0.78)					
Antisocial personality disorder								
	yes (*n* = 245)	45 (0.48)	200 (0.33)	0.003**	1.94 (1.25–3.01)	0.003**	0.76 (0.37–1.53)	0.437
	no (*n* = 462)	48 (0.52)	414 (0.67)					

*SAD* Social Anxiety Disorder, *OCD* Obsessive–compulsive disorder, *PTSD* Posttraumatic stress disorder, *GAD* General anxiety disorder, *OR* Odds Ratio, *CI* Confidence Interval–

*p < 0.05, **p < 0.01, ***p < 0.001

### Comparison with other studies

Suicide risk in prison in metropolitan France has also been assessed using the MINI. In mainland France 40.3% (322/799) of male prisoners had a suicide risk, in Martinique the risk was estimated to be 26% (26/100). Among female prisoners in metropolitan France 61% (61/99) had a suicide risk.

## Discussion

The present study revealed that 13.2% of the inmates had a suicidal risk, 14% of which had a high risk. Risk factors included being sentenced for sexual abuse, being male, smoking, childhood abuse and psychiatric disorders such as depression, dysthymia, panic disorder and GAD. Compared to mainland France and Martinique, the prevalence was lower in French Guiana (Tables 3 and 4). It is not clear why the risk for suicide was lower. Perhaps, the high proportion of foreign detainees leads to an incarcerated population that often does not have strong community ties in French Guiana and thus is less subjected to social stigma. The frequency of drug trafficking in this transit region also presumably trivializes it and makes it less stigmatizing than in France. However, it is noteworthy that whereas the risk of suicide was relatively low, the actual average suicide rate was not significantly different than in mainland France [27]. In our study population, we found similar risk factors to those identified by the WHO and in previous studies [28]. After a Pubmed search for suicidality in prisons, we found just one study conducted in a Colombian prison [29] with 14.9% of the inmates with a high suicide risk, with the highest rates among single inmates under the age of 30, those who previously attempted suicide and suffering from domestic violence. In England and France the study of predictors showed that psychiatric illnesses were associated with the risk of suicide, being primordial because of the high prevalence of mental disorders among detainees [23, 30]. The amplification of suicidality by psychiatric comorbidity in mood-disordered patients in general populations has been proven in several studies [31]. French Guianese prisoners with major depressive episodes had a high suicide risk, thus highlighting the need to identify them early given the temporal dynamics of the risk of suicide during incarceration [32]. Generalized anxiety is important to identify because it is a precursor in a number of actual suicides [33]. Little is known about the relationship between panic disorders and suicide in general and even less is known in the context of prison. Even if it is the subject of a long and controversial debate [31, 34], the impact of panic disorder on the severity of depression and the increased likelihood of suicide have been shown in the general population [35, 36]. We found a similar association with a two-fold increase in suicide risk for subjects. As described elsewhere, the incarceration motive was associated with differences in suicidal risk [37, 38]. In mainland France, actual suicide was associated with sentences for harm to individuals, such as violence or homicide, and sexual assault [37]. In our study, having been sentenced for sexual assault or rape remained significantly associated with suicide risk with an OR of 5.98 in the final model. Although it was no longer significant in the multivariate analysis, those sentenced for drug trafficking seemed associated with a lower suicide risk. Drug trafficking (34.4%) was the most frequent crime for those with and without a suicide risk in our study population and it is the only source of income for many. It might thus be more socially acceptable in parts of society and prison, and thus seen as a transient incident. But rape and sexual abuse are considered as major moral transgressions and are stigmatised in society and also in prison. There were no prisoners within those incarcerated for rape or sexual assault who had been abused themselves. Being a victim of a sexual trauma or abuse during childhood was a highly significant risk factor, especially for men. Several studies showed the association of childhood abuse and suicidality, and conclude that targeting this population at risk should be a major prevention measure [39–41]. This phenomena has been confirmed by Ystgaard et al. when investigating repeated suicidal behaviour, but due to its complexity the entire mechanism is not yet fully understood [42]. In addition, the influence of childhood trauma on suicidal behaviour in inmates has been associated with the early onset of this behaviour, psychiatric disorder and destructive personality [43]. In an American study, childhood trauma was identified as an independent factor for female inmates but was not associated with substance abuse, mental disorders or incarceration duration [44]. This thus supports the hypothesis of an actual distress situation rather than a long-term condition. While smoking and substance abuse seemed to be associated with suicidal risk, alcohol consumption however was not. These variables have already been associated previously with suicide risk [7, 45]. Although young males have been reported to have a higher suicidal risk [37], we did not find a correlation between age and gender in our study population. For women (58%) as for men (38%) the majority of persons with a suicide risk were indeed those between 20 and 30 years of age but these results were not statistically significant.

**Table 3:** Comparison of prevalence of suicide risk of men in French Guiana between metropolitan France and Martinique

Suicide risk			
	n	%	*p*-value
French Guiana, 2014 (*n* = 647)	81	12.5	
Metropolitan France, 2004 (*n* = 799)	322	40.3	<0.0001***
Martinique, 2004 (*n* = 100)	26	26.0	<0.001**

**p < 0.01, ***p < 0.001

**Table 4:** Comparison of prevalence of suicide risk of women in French Guiana between metropolitan France and Martinique

Suicide risk			
	n	%	*p*-value
French Guiana, 2014 (*n* = 60)	12	20.0	
Metropolitan France, 2004 (*n* = 99)	61	61.0	<0.0001***

***p < 0.001

Characteristics, which we had intuitively predicted to affect suicidal risk, such as profession, early loss of relatives, family status, separation of parents in childhood, were not associated with suicidal risk in our study population. This multicultural population is often assumed to have very important differences. However, here there was no obvious difference between nationalities. What mattered was not culture but psychiatric disorders.

The study limitations were that it is declarative data in a context of incarceration which may have led a biased estimate. Some of the inmates refusing to answer may have had a different suicide risk than those who accepted. There have been debates about the necessity to formally validate suicide risk assessment tools [46]. The MINI scale has limitations failing to identify some cases of suicidality (false negatives). Although, a thorough evaluation of its validity would require further prospective studies, it has shown its interest in a prospective study [26].

The cross sectional design was also a limiting aspect since the risk varies in time after the initial incarceration shock. Despite these drawbacks, this was nevertheless a study using the same tools as the one conducted in France, which allowed to show the lower suicide risk in prison in French Guiana relative to France.

## Conclusion

This is the first study to measure the prevalence and the predictors of suicidal risk in the only French Guianese prison. To our knowledge, there are very few studies relative to suicidality in prison in South America or in the Caribbean. An initial assumption was that the multicultural South American context may have uncovered possibilities of risk stratification by nationality, a hypothesis that is refuted by the present results. Associations of suicide risk were found with mental disorders like depression, dysthymia, panic disorder and anxiety, males abused in childhood, smoking and being sentenced for sexual abuse. It gives a snapshot of the situation that will help improve the prevention of morbidity and mortality in prison, as required by French law since 2000. Longitudinal follow-up should further study time dependent aspects like the first incarceration shock and subsequent adaptation, and the influence of events within the prison such as other suicide attempts.

## Abbreviations

CI: Confidence Interval
DSI: Diagnostic Structured Interview
DSM-V: Diagnostic and Statistical Manual V
GAD: General Anxiety
INSERM: Institut national de la santé et de la recherche médicale
IRB: Institutional Review Board
MINI: Mini International Neuropsychiatric Interview
OR: Odds Ratio
UCSA: Unité de consultation et de soins ambulatoires
UFPI: Unité fonctionnelle de psychiatrie intra-carcérale
WHO: World Health Organisation
